# Non-Steroid Anti-Inflammatory Drugs Are Better than Acetaminophen on Fever Control at Acute Stage of Fracture

**DOI:** 10.1371/journal.pone.0137225

**Published:** 2015-10-02

**Authors:** Kuang-Ting Yeh, Wen-Tien Wu, Yi-Maun Subeq, Chi-Chien Niu, Kuang-Wen Liao, Ing-Ho Chen, Jen-Hung Wang, Ru-Ping Lee

**Affiliations:** 1 Institute of Medical Sciences, Tzu Chi University, Hualien, Taiwan, R.O.C.; 2 Department of Orthopedics, Hualien Tzu Chi Hospital, Buddhist Tzu Chi Medical Foundation, Hualien, Taiwan, R.O.C.; 3 School of Medicine, Tzu Chi University, Hualien, Taiwan, R.O.C.; 4 Department of Nursing, Tzu Chi University, Hualien, Taiwan, R.O.C.; 5 Department of Orthopaedic Surgery, Chang Gung Memorial Hospital, Taoyuan, Taiwan, R.O.C.; 6 Department of Biological Science and Technology, National Chiao Tung University, Hsin-Chu, Taiwan, R.O.C.; 7 Department of Research, Hualien Tzu Chi Hospital, Buddhist Tzu Chi Medical Foundation Hualien, Taiwan, R.O.C.; Boston Children’s Hospital and Harvard Medical School, UNITED STATES

## Abstract

In addition to adequate surgical fixation and an aggressive rehabilitation program, pain relief is one of the most critical factors in the acute stage of fracture treatment. The most common analgesics are nonsteroid anti-inflammatory drugs and Acetaminophen, both of which relieve pain and reduce body temperature. In clinical experiences, they exhibit effective pain control; however, their influence on body temperature remains controversial. This study is aimed at determining the effects of analgesics at the acute stage of traumatic fracture by performing a clinical retrospective study of patients with fractures and a fracture animal model. The retrospective study revealed that, in the acetaminophen group, the mean value of postmedication body temperature (BT) was significantly higher than that of the premedication BT. The change in BT was highly related with the medication rather than other risk factors. Forty eight 12-week-old male Wistar rats were divided into 6 groups: a control group, fracture group, fracture-Acetaminophen group, Acetaminophen group, fracture-Arcoxia group, and Arcoxia group. Fracture rats were prepared by breaking their unilateral tibia and fibula. Their inflammation conditions were evaluated by measuring their serum cytokine level and their physiological status was evaluated by estimating their central temperature, heart rate, and mean blood pressure. The hepatic adverse effects were assessed by measuring the serum levels of aspartate aminotransferase (sGOT) and alanine aminotransferase (sGPT). The central temperature in the fracture-Acetaminophen group exceeded that in the groups fed normal saline water or Arcoxia. Accumulated hepatic injury was presented as steadily ascending curves of sGOT and sGPT. Inflammation-related cytokine levels were not higher in the Acetaminophen fracture group and were significantly lower in the fracture-Arcoxia group. Fever appeared to be aggravated by acetaminophen and more related to the elevation of hepatic enzymes than to the change in the inflammation-related cytokines. We suggest that acetaminophen may aggravate fever at the acute stage of fracture. This response is highly related to the accumulated and exacerbated side effects of hepatitis that are caused by the medication and trauma.

## Introduction

Fracture is a common cause of admission of orthopedic patients. In addition to adequate surgical fixation and an aggressive rehabilitation program, pain relief is one of the most critical factors in the acute stage treatment of trauma. The most common analgesics are nonsteroid anti-inflammatory drugs (NSAIDs) and acetaminophen. NSAIDs are well-known as effective pain control agents that reduce the inflammation stress caused by trauma [1], which is associated with the release of proinflammatory mediators and the priming of circulating neutrophils [2]. The mechanism of the analgesic action of acetaminophen remains incompletely understood, particularly at the acute stage of trauma [3]. Acetaminophen is likely a factor in reducing a ferryl protoporphyrin IX radical cation (Fe4+ = OPP*+) within the peroxidase site of prostaglandin endoperoxide H synthase (PGHS) enzyme [3]. In turn, the Fe4+ = OPP*+ generates tyrosine radicals in the place of PGHS cyclooxygenase (COX), which are essential catalyzing AA oxidation reactions [4, 5]. Although acetaminophen-induced hepatotoxicity remains a significant public health concern, acetaminophen is widely used as an analgesic for fractured and postoperative patients because of its weaker gastrointestinal side effects than NSAIDs and the improved compliance of patients taking adequate doses in the short term [6, 7].

Elevated body temperature (BT) may be related to the inflammation of acute trauma. The proinflammatory cytokines of trauma include tumor necrosis factor-alpha (TNF-α), interleukin (IL)-6, and IL-10. In murine models, TNF-α and IL-6 are expressed at the fracture site within 24 hour (h) of injury [8]. The expression of TNF-α in a fracture follows a biphasic pattern and peaks during the initiation of fracture repair, followed by a second peak upon the transition from chondrogenesis to osteogenesis during endochondral maturation [9]. Trauma has previously been demonstrated to influence IL-10 levels, which were known as containing both proinflammatory and anti-inflammatory characteristics, and their responses revealed as highly related to injury severity and mortality in the early phase of trauma [10]. As a pleiotropic cytokine produced by both T cells and macrophages, it possesses both anti-inflammatory and immunosuppressive properties. However, IL-10 also has immunostimulatory properties on T cells and NK cells to increase IFN-γ production. Previous studies reveal that increased concentrations of IL-10 have been associated with poor clinical results [11]. Endogenous IL-10 production may also decrease T-cell apoptosis, exacerbate T-cell dysfunction, and increase mortality in some bacterial models of sepsis or after thermal injury [12]. In addition, BT alteration is related to the elevated serum cytokine or to the side effects of acetaminophen use at the acute stage of trauma. Few recent studies have investigated the analgesics’ effects on change of BT. In this study, an animal model was designed to observe how acetaminophen influences the acute stage of fracture.

After a patient with fracture trauma receives internal fixation surgery, the most common clinical complaints are pain in the affected limb and elevated BT. The most commonly used analgesics in such scenarios are acetaminophen and NSAIDs. We have found in clinical experience that both these analgesics are effective; however, their influences on BT appear to vary. Acetaminophen also appeared to exhibit poor BT downregulation in the trauma patients, in contrast to its effects as a clinical antipyretic agent. Therefore, to resolve this problem, we retrospectively collected the BT data of patients with fractures. Through the retrospective study of these patients and the fracture animal model, this study is aimed to determine the antipyretic effects of different analgesics at the acute stage of traumatic fracture.

## Materials and Methods

### Clinical study data collection

The BT data of patients with fractures admitted to our hospital were collected from 2012 to 2013. The data were recorded before and after acetaminophen or Arcoxia (a kind of NSAID) administration. The inclusion criteria were that the patients needed to have 1) at least one limb fracture and have undergone surgery in our hospital, 2) been administered acetaminophen at 500 mg or Arcoxia at 60 mg after the injury, and 3) their BT data recorded before the analgesic being administered and 4 hs after medication. The exclusion criteria were that patients could not have 1) been administered with multiple analgesics before or between the BT estimation points, 2) had injuries other than orthopedic trauma, or 3) had comorbidities of poorly controlled medical conditions. We also collected age, sex, and fracture site data.

### Animal experiment preparation

The Animal Use Protocol Board of Tzu-Chi Hospital approved this study (IACUC Approval No.101-18). Forty-eight male Wistar–Kyoto rats, aged 12 to 15 weeks and weighing 300 to 320 g were used in the study. The rats were purchased from the National Laboratory Animal Center (Taipei, Taiwan) and housed in the Tzu-Chi University Animal Center. The room temperature was maintained at 22°C ± 1°C and a 12-h light/dark cycle was maintained. Food and water were provided ad libitum. The rats were anesthetized by ether inhalation for approximately 10 minute (min). While each rat was under anesthesia, a polyethylene-50 catheter was inserted into the right femoral artery and vein according to aseptic surgical techniques. The femoral venous catheter was prepared for medicine administration. The femoral arterial catheter was connected with a 3-way adaptor. One end was connected to record the arterial blood pressure (BP) and heart rate (HR) on a recorder (Power Lab, AD Instruments, Mountain View, CA); the remaining end was prepared to take blood samples. The catheterization was completed and the wound was sutured within 10 mins. A left tibia and fibula fracture was made in less than 10 second (sec) by using the 3-point bending method [13]. The fractured limb was fixed using an external wood stick wrapped in elastic bandaging. The procedures from catheterization to 3-point bending fracture took 15 mins in average, and the rats were under ether anesthesia in this duration. After the procedures, each animal was placed in a metabolic cage. The rats were kept in the cages during the experiment and their physiologic changes were monitored continuously for 48 hs [14].

### Animal classification

The animals were divided into the control (sham) group, Arcoxia (Non-Fr+Arcoxia), Acetaminophen (Non-Fr+Acetaminophen), fracture (Fr), and fracture with Arcoxia (Fr+Arcoxia) or Acetaminophen (Fr+Acetaminophen) administration groups. Each group comprised 8 animals. After completing catheterization, left tibial and fibular shaft fractures were performed using the 3-point bending method within 10 secs [13] in the Fr, Fr+Arcoxia, and Fr+Acetaminophen groups under anesthesia of ether. The fractured limb was fixed using an external wood stick wrapped in elastic bandaging. Acetaminophen (Anti-phen Syrup, Center) and Etoricoxib (Arcoxia, MSD) were administered twice a day by oral gavages in doses of 16.67 mg/kg and 1 mg/kg, respectively, diluted with normal saline to 1 mL. The dosages of acetaminophen and Arcoxia administration are according to the human dose for a 60 kg adult. The control and fracture groups were also fed with desterilized water 1 mL twice a day. All drug administration groups were fed by the same as people at a fixed time.

### Blood sample measurements

Blood samples were collected as the baseline values (as point “pre” on the figure) before fractures for all Fr groups and before administration of normal saline or drugs in all nonfracture groups. The blood sample measurements included levels of serum aspartate aminotransferase (sGOT), alanine aminotransferase (sGPT), creatine phosphokinase (CPK), and lactate dehydrogenase (LDH). The samples were taken before the fracture procedure and 1, 3, 6, 9, 12, 18, 24, 36, and 48 hs after the fractured limb was fixed. The 0.8 ml blood was taken at each time point and equally amount of normal saline was infused after blood withdrew. The samples were centrifuged at 3000 rpm at 4°C for 10 min, then decanted the plasma and separated it into 2 parts. One part was stored at 4°C for biochemical analysis of its sGOT, sGPT, CPK, and LDH levels with an auto analyzer (Cobas Integra 800, Roche Diagnostics, Basel, Switzerland) and the other part was stored at –80°C for later measurement of TNF-α, IL-6, and IL-10 concentration.

### Data analysis

The SPSS software package, Version 13.0, was used for statistical analysis. To assess statistical significance, an unpaired Student’s *t* test was performed for the clinical study. The level of significance was set at p<0.05 to compare the preoperative and postoperative data. Age, sex, and sites of fracture were set as the independent variables of BT change for the clinical data. To determine the independent variable with the greatest impact, a stepwise generalized linear model was used. The final selection of the independent variables was determined according to the adjusted R-square value. A statistical analysis was then performed on each standard regression coefficient of the independent variables. Those variables with a significance of p<0.05 were selected as the factors influencing BT change. The magnitude of the impact was defined as the value of the standard regression coefficient for each factor.

The animal study data were expressed as mean±standard error of mean. A two-way analysis of variance (ANOVA) was used to calculate the statistical differences between specific time points. Differences in mean values at the same time points between groups were analyzed using Duncan’s post hoc multiple comparisons. The relationship between plasma TNF-α, IL-6, and IL-10 was analyzed using Spearman’s rho correlation. A level of p<0.05 (2-tailed) was considered significant.

## Results

### Clinical study data

A total of 135 cases were collected, comprising 62 men and 73 women (Table 1). Among them, 91 patients received acetaminophen and 44 received Arcoxia 4 hs after fracture occurred. They were divided into Acetaminophen group and Arcoxia group. The patients had a mean age of 53.1 years and more than half were aged over 60 years and had a single fracture site. There were no differences between the demographic data of these two groups, including age, sex distribution ratio, number and location of fracture sites, and premedication BT. In Acetaminophen group, the mean postmedication BT was significantly higher than that of mean premedication BT. The mean postmedication BT of Acetaminophen group was also higher than that of Arcoxia group. The regression analysis of generalized linear model revealed that the elevated BT was not influenced by older age, sex, number of fracture sites, or fracture site location (Table 2).

**Table 1:** Demographic Data of Patients (n = 135).

Items	Acetaminophen (n = 91)	Arcoxia (n = 44)	p-value
**Age**	54.1±25.1	52.1±17.9	0.777
**Age Group**			0.784
≦50 y/o	36(39.6%)	16(36.4%)	
>50 y/o	55(60.4%)	28(63.6%)	
**Gender**			0.263
Female	54(59.3%)	19(43.2%)	
Male	37(40.7%)	25(56.8%)	
**More than one fracture site**			0.692
No	79(86.8%)	40(90.9%)	
Yes	12(13.2%)	4(9.1%)	
**Fracture site**			0.052
Upper limbs	38(41.8%)	34(77.2%)	
Lower limbs	48(52.7%)	9(20.5%)	
Upper and lower limbs	5(5.5%)	1(2.3%)	
**Pre-medication BT**	36.5±0.4	36.7±0.4	0.307
**Post-medication BT**	37.0±0.5	36.2±0.2	<0.001
**ΔBT**	0.5±0.4	-0.4±0.3	<0.001

**Table 2:** Factors Associated with the Change of Body Temperature (n = 135).

Items	Regression Coefficient	95% CI	p-value
**Age Group**			
≦50 y/o	0.057	(-0.119, 0.234)	0.522
>50 y/o	References	References	NA
**Gender**			
Female	0.102	(-0.069, 0.272)	0.239
Male	References	References	NA
**More than one fracture site**			
No	-0.051	(-0.361, 0.260)	0.747
Yes	References	References	NA
**Fracture site**			
Upper limbs	-0.615	(-1.079, -0.151)	0.053
Lower limbs	-0.437	(-0.931, 0.056)	0.082
Upper and lower limbs	References	References	NA
**Medication**			
Acetaminophen	0.744	(0.496, 0.993)	<0.001
Arcoxia	References	References	NA

Dependent variable: Change of body temperature. NA: Not Apply.

### Animal experiment data

**Fig 1:**
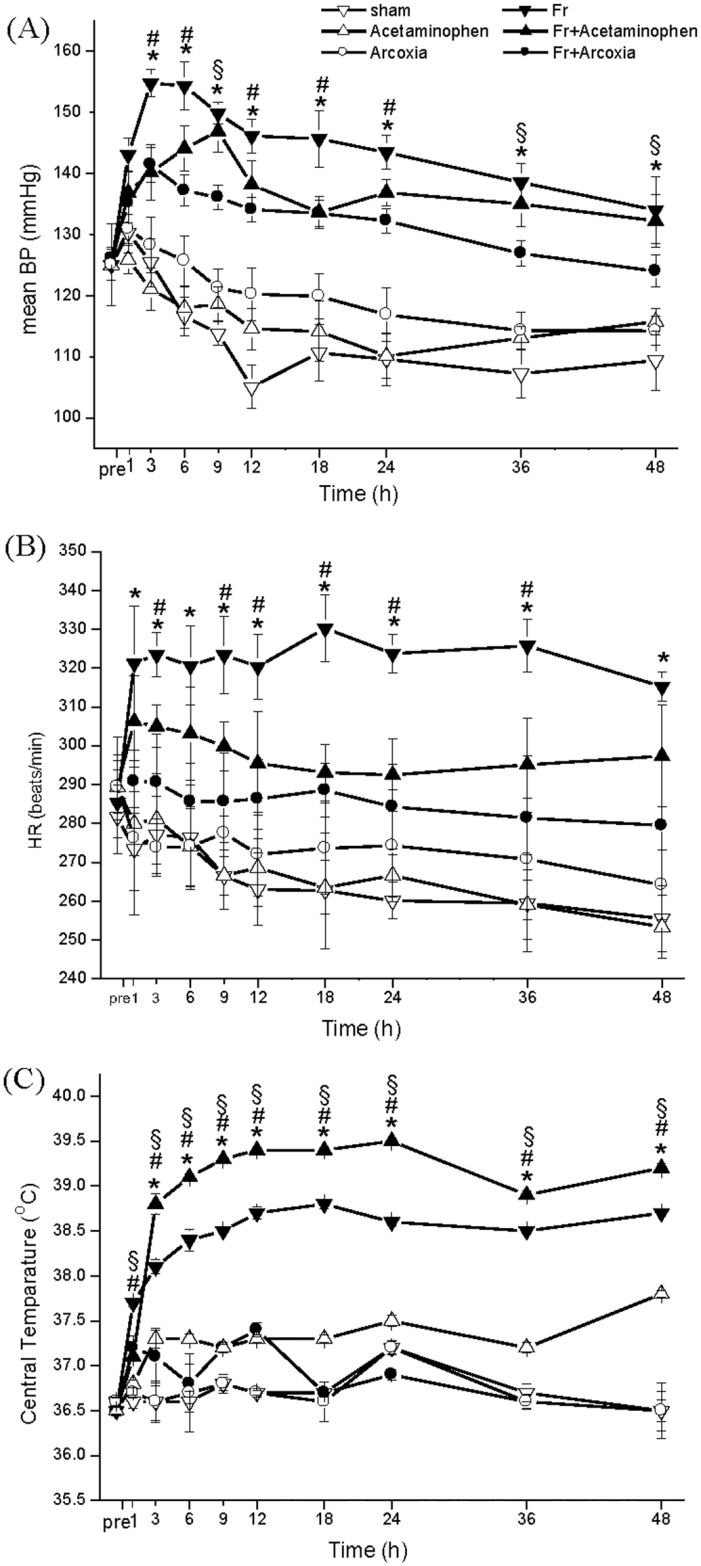
The mean values of blood pressure (A), heart rate (B), and central body temperature (C) of the rats. The central temperature in the Fr+Acetaminophen group was higher than the Fr+Arcoxia group. Two-way ANOVA with Duncan’s post hoc multiple comparisons was used. *p<0.05 for the Fr group compared with the Fr+Arcoxia group. ^#^p<0.05 for the Fr group compared with the Fr+Acetaminophen group. ^§^p<0.05 for the Fr+Arcoxia group compared with the Fr+Acetaminophen group.

All the six rat groups appeared in high consistency of physiologic conditions before fracture. Their mean blood pressure ranged from 120 to 130 mmHg and heart rate ranged from 280 to 290 beats per min (b.p.m) (Fig 1A and 1B). After fracture, the Fr group had higher values of BP and HR than those in other groups. The Fr+Arcoxia group had a significantly decreasing in BP (Fig 1A) and HR (Fig 1B) than Fr group from 3 to 48 hs after fracture. The Fr+Acetaminophen group had a lower BP than Fr group at 3, 6, 12, 18, and 24 h (Fig 1A) after fracture. The heart rates of fractured rats were mildly lower after being fed Acetaminophen than the Fr group (Fig 1B). The mean BTs were significantly higher in the fracture group fed with Acetaminophen at all the time points after fracture than them of other 5 groups (Fig 1C). The second highest central temperature appeared in the fracture group. On the other hand, the mean BTs of Fr+Arcoxia group was maintained in normal range after fracture.

**Fig 2:**
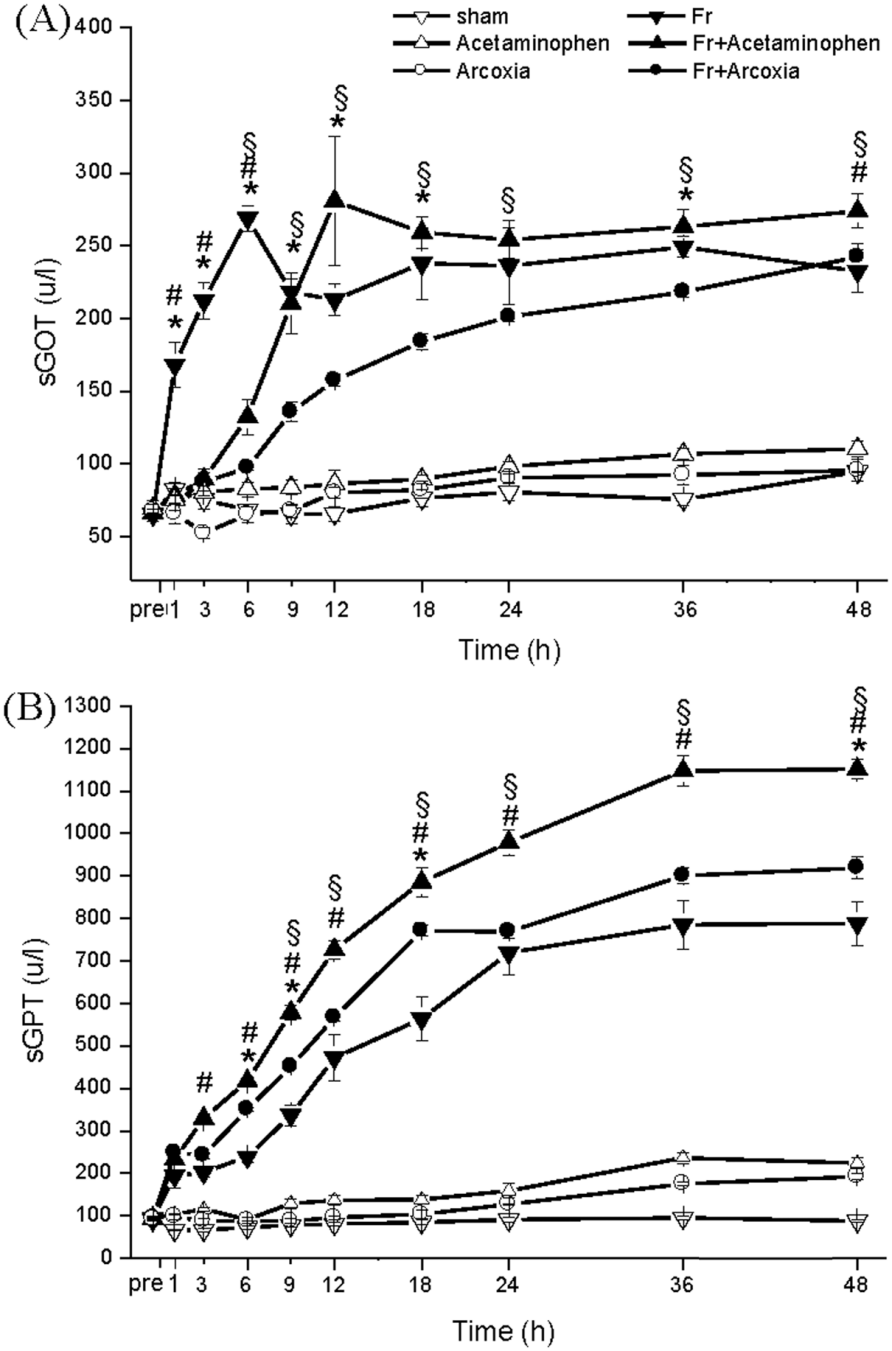
Influence on liver function by injury and different medications. The serum levels of sGOT (A) and sGPT (B) in the Fr+Arcoxia group were lower than those in the Fr+Acetaminophen group after fracture. Two-way ANOVA with Duncan’s post hoc multiple comparisons was used. *p<0.05 for the Fr group compared with the Fr+Arcoxia group. ^#^p<0.05 for the Fr group compared with the Fr+Acetaminophen group. ^§^p<0.05 for the Fr+Arcoxia group compared with the Fr+Acetaminophen group.

Serum hepatic enzyme levels were higher in the fracture groups than in the nonfracture groups (Fig 2A and 2B). Fr+Acetaminophen group appeared to elevate both sGOT (Fig 2A) and sGPT (Fig 2B) levels in accumulated curves. Fr+Arcoxia group exhibited lower sGOT and sGPT levels than Fr+Acetaminophen group after fracture.

**Fig 3:**
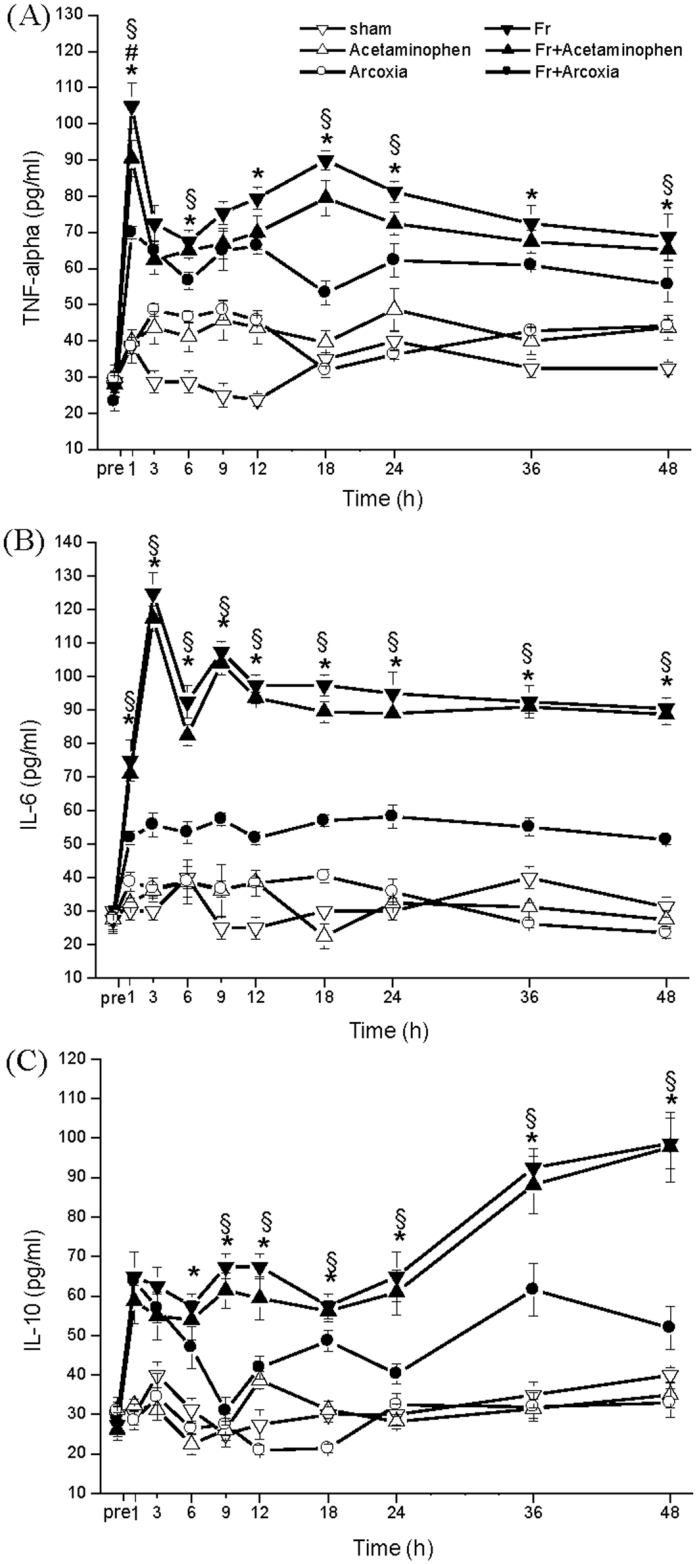
Influence on inflammation related cytokines TNF-alpha (A), IL-6 (B), and IL-10 (C) by injury and different medications. Inflammation-related cytokine levels were significantly lower in the Fr+Arcoxia group. Two-way ANOVA with Spearman’s rho correlation was used.*p<0.05 for the Fr group compared with the Fr+Arcoxia group. ^#^p<0.05 for the Fr group compared with the Fr+Acetaminophen group. ^§^p<0.05 for the Fr+Arcoxia group compared with the Fr+Acetaminophen group.

Levels of TNF-α, IL-6, and IL-10 exhibited the same distribution curve in both Fr and Fr+Acetaminophen groups. The serum TNF-α level elevated rapidly in the early phase of the fracture and then returned to a regular level and stabilized (Fig 3A). The serum IL-6 level also increased quickly in the early phase (Fig 3B). The serum IL-10 level elevated in the early phase and then increased again 18 h after fracture (Fig 3C). The peak after 1 h of the serum TNF-α level was significantly reduced in Fr+Arcoxia group. The serum IL-6 level at every time point was lower in Fr+Arcoxia group than in Fr and Fr+Acetaminophen groups.

**Fig 4:**
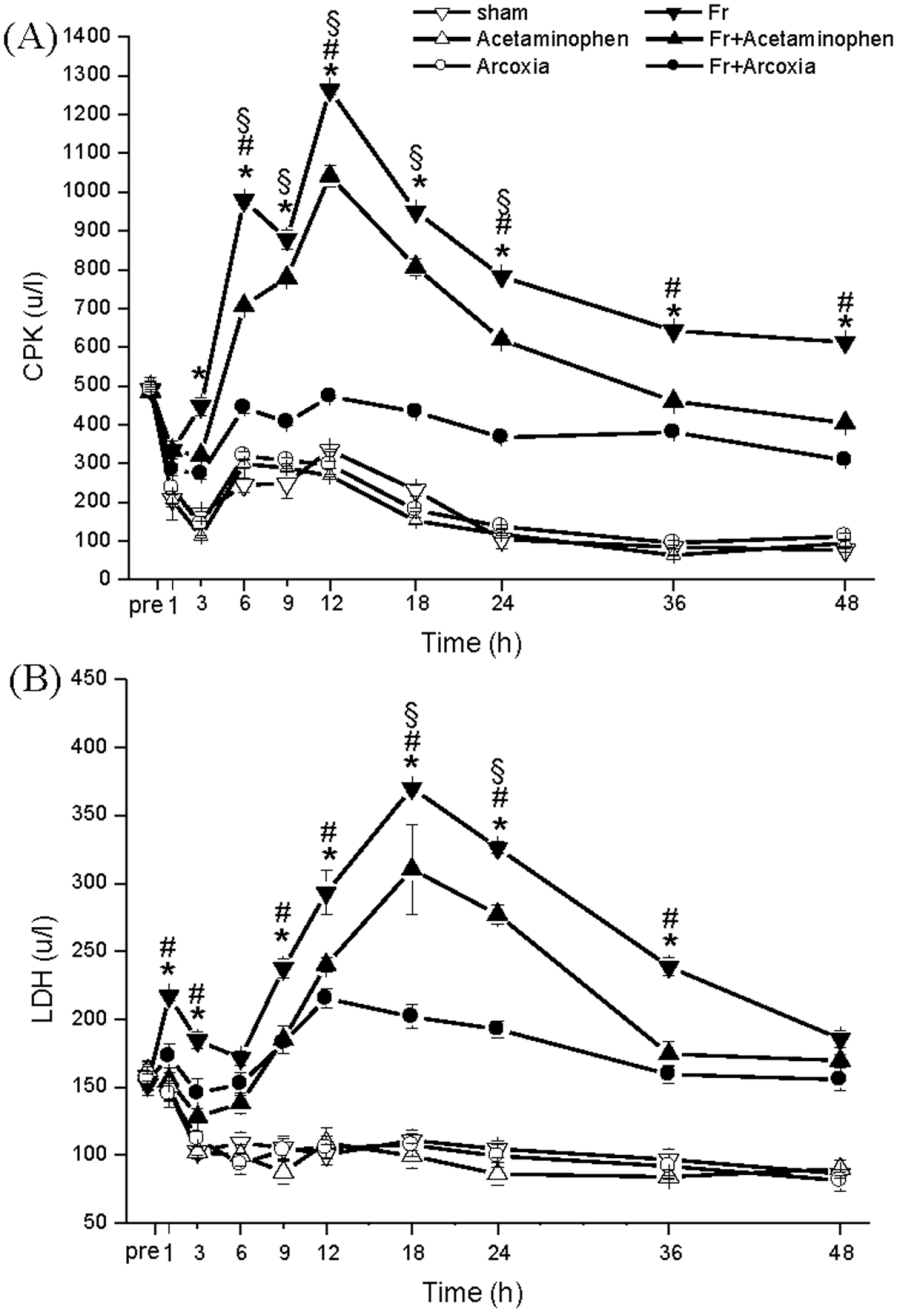
Influence on soft-tissue injury related enzymes by limb fracture and different medications. In the Fr+Arcoxia group, the serum levels of CPK (A) and LDH (B) were significantly lower than those in Fr group and Fr+Acetaminophen group. Two-way ANOVA with Duncan’s post hoc multiple comparisons was used. *p<0.05 for the Fr group compared with the Fr+Arcoxia group. ^#^p<0.05 for the Fr group compared with the Fr+Acetaminophen group. ^§^p<0.05 for the Fr+Arcoxia group compared with the Fr+Acetaminophen group.

Soft tissue injury related enzyme, serum CPK (Fig 4A) and LDH (Fig 4B), levels in Fr+Acetaminophen group were generally mild, lower than in the Fr group, and revealed similar distribution curves. However, in Fr+Arcoxia group, the serum CPK and LDH levels were significantly lower than those in Fr group and Fr+Acetaminophen group.

## Discussion

Pain relief by administering acetaminophen every 6–8 h is common during fracture surgery and care. Taking vital signs every 6–8 h is also a crucial nursing care principle in treating these patients. Administering analgesics appears to cause changes in BT. We compared BT before and after the administration of one oral dose of Acetaminophen or Arcoxia and analyzed the initial risk factors of BT change. We then designed an animal study using a conscious rat model with unilateral tibial fracture. The data showed that the patients who received Acetaminophen had significantly higher BTs than they had preadministration. The postadministration BTs in the Acetaminophen group were higher than those in the Arcoxia group. Regression analysis revealed that neither age, nor sex, nor fracture condition significantly affected BT elevation. These data mean that administering different types of analgesics is the most critical factor in BT change. However, we cannot compare the results with a group that did not receive analgesics because no patients with fractures can tolerate the pain without analgesic administration. This limitation is why we used an animal model to confirm our findings.

Thermoregulation can be influenced by numerous internal and external environmental factors through the regulation of a set point that is controlled by the hypothalamus. In nontrauma patients, such as those with an infection, circulatory pyrogens caused by bacteria or a virus upregulates the set point and cause fever [15]. Acetaminophen can decrease the BT in nontrauma patients by reversing these reactions that cause fever. Acetaminophen yields its antipyretic effect by regulating the set point and through weak anti-inflammation effects. However, in our animal study, the fractured rats fed with acetaminophen exhibited higher central temperatures throughout the experimental period. The proinflammatory cytokine levels of Fr and Fr+Acetaminophen groups did not vary significantly. These results revealed that acetaminophen exerted no obvious effect in reducing central temperature in the acute stage of traumatic fracture. Fractures could cause local inflammation and stimulate inflammatory cytokines formation, which could cause local soft tissue swelling, pain, and low-grade fever. According to the decreasing of heart rates, acetaminophen may have inhibited pain in the fractured rats, however, did not suppress the inflammation reaction according to the increasing of serum TNF-α, IL-6, and IL-10 levels. Under this interference effect of acetaminophen, central temperatures could be elevated to abnormally high levels. Considering this phenomenon, the role of acetaminophen in patients with severe local inflammation should be reevaluated.

According to the time course of fever relapse, hepatic enzymes are elevated significantly at the same time as the fever. The fever appears to be closely related to the incidence of hepatitis. Acetaminophen appeared to elevate hepatic-related enzymes such as sGOT and sGPT, as did fracture stimulation. This phenomenon might be related to acute hepatic injury. Animal models of Acetaminophen hepatotoxicity have been well documented in other studies [16, 17]. Fracture-induced inflammation and soft tissue injury can also cause acute-stage hepatic injury through inflammation and vasodilation effects [18, 19]. Our results demonstrate the vasodilatory response from the time course of mean blood pressure, with lower values noted in both fracture groups. An accumulative injury effect was noted from the time course of sGOT and sGPT in the fracture group fed with acetaminophen. This phenomenon may occur because receiving acetaminophen for more than 48 hs exacerbated liver injury in the patients with fractures. Serum creatinine levels appeared normal in all 6 groups, suggesting that renal injury may be prevented by adequate hydration and safe micturition. Furthermore, renal damage may not occur under general doses of acetaminophen administration at the acute stage of fracture. Our results reveal that Arcoxia reduces the effect of inflammation-related cytokines and lowers BT levels at every time point at the acute stage. Arcoxia is one of the most common COX-2-inhibitor NSAIDs. Compared with conventional COX inhibitors, selective COX-2 inhibitors do not influence the platelet function; therefore, they may be safer by preventing perioperative bleeding [20]. Administered preoperatively, COX-2 inhibitors appear to reduce postoperative pain efficiently, according to recent clinical studies [21]. The inhibitors suppressed pain by reducing the serum inflammatory cytokines, which can concomitantly facilitate the decrease of hyperinflammation caused by acute fracture injury [22].

In this study, we focus on the influence of therapeutic dose of two different kinds of analgesics on acute stage of fracture instead of antipyretic effect of them. In previous animal study, the oral doses of antinociceptive properties of acetaminophen ranged from 10 to 300 mg/kg [23]. On the other hand, the oral dose for antipyretic effects ranged from 20 to 200 mg /kg [24–26]. We give an oral dose as acetaminophen 16.67 mg/kg for the rats according to the clinic therapeutic dose for pain relief. From the data we can see that mean heart rate of fracture treated with acetaminophen group was lower than fracture group at all the time points. This result implied that acetaminophen relives pain of the fractured rats effectively. However, the antipyretic effect is not obvious and it could cause of the lower oral doses of acetaminophen. In the other way, arcoxia was also given according to human therapeutic dose as a pain relief agent (1 mg/kg). The fracture treated with arcoxia group has significantly lower mean central temperature than fracture group at all the time points. The data showed a different effect on central temperature between two different kinds of analgesics that is one of our meaningful findings. Higher dose of acetaminophen is often used for antipyretic effect and may aggravate hepatocellular injury, and it's not suitable for pain relief of trauma-related fracture. In the future study, the higher oral dose of acetaminophen should be administered to focus on its obvious antipyretic effect on fracture condition. In addition, the level of hepatic damage should be included and further evaluated.

The effect of acetaminophen in the acute stage of injury has seldom been studied. Elevated BT after acetaminophen administration was observed in our clinical retrospective study. In our animal experiment, the time course of physiological status, hepatic and renal function, and inflammatory cytokines were examined. The data revealed that acetaminophen may aggravate fever in the acute stage of fracture. This condition is highly related to the accumulated and exacerbated side effects of hepatitis, which are caused by the medication and trauma. We suggest that the selection of analgesics and combined protection methods should be considered carefully at this stage of fracture.
